# A case of malignant insulinoma responsive to somatostatin analogs treatment

**DOI:** 10.1186/s12902-018-0325-4

**Published:** 2018-12-27

**Authors:** Mariasmeralda Caliri, Valentina Verdiani, Edoardo Mannucci, Vittorio Briganti, Luca Landoni, Alessandro Esposito, Giulia Burato, Carlo Maria Rotella, Massimo Mannelli, Alessandro Peri

**Affiliations:** 1 Endocrine Unit, Department of Experimental and Clinical Biomedical Sciences “Mario Serio”, University of Florence, Careggi University Hospital, Florence, Italy; 2 Diabetology Unit, Department of Experimental and Clinical Biomedical Sciences “Mario Serio”, University of Florence, Careggi University Hospital, Florence, Italy; 3 Division of Nuclear Medicine, Careggi University Hospital, Florence, Italy; 4 General and Pancreatic Surgery Department, The Pancreas Institute-University of Verona Hospital Trust, Verona, Italy; 5 Department of Pathology and Diagnostics, University of Verona Hospital Trust, Verona, Italy

**Keywords:** Insulinoma, Hypoglycemia, Somatostatin analogs, Octreotide

## Abstract

**Background:**

Insulinoma is a rare tumour representing 1–2% of all pancreatic neoplasms and it is malignant in only 10% of cases. Locoregional invasion or metastases define malignancy, whereas the dimension (> 2 cm), CK19 status, the tumor staging and grading (Ki67 > 2%), and the age of onset (> 50 years) can be considered elements of suspect.

**Case presentation:**

We describe the case of a 68-year-old man presenting symptoms compatible with hypoglycemia. The symptoms regressed with food intake. These episodes initially occurred during physical activity, later also during fasting. The fasting test was performed and the laboratory results showed endogenous hyperinsulinemia compatible with insulinoma. The patient appeared responsive to somatostatin analogs and so he was treated with short acting octreotide, obtaining a good control of glycemia. Imaging investigations showed the presence of a lesion of the uncinate pancreatic process of about 4 cm with a high sst2 receptor density. The patient underwent exploratory laparotomy and duodenocephalopancreasectomy after one month.

The definitive histological examination revealed an insulinoma (T3N1MO, AGCC VII G1) with a low replicative index (Ki67: 2%).

**Conclusions:**

This report describes a case of malignant insulinoma responsive to octreotide analogs administered pre-operatively in order to try to prevent hypoglycemia. The response to octreotide analogs is not predictable and should be initially assessed under strict clinical surveillance.

## Background

Insulinoma is a rare tumour representing 1–2% of all pancreatic neoplasms [1]. It is malignant in only 10% of cases [2]. The malignancy can be stated only in the presence of locoregional invasion into the surrounding soft tissue, lymph node or liver metastases [3]. The dimension > 2 cm, CK19 status, the tumor staging and grading (Ki67 > 2%), and the age of onset > 50 years can be considered indicators of malignancy [4–6]. However, in literature, there are some reports where about 40–50% of malignant insulinomas are < 2 cm [7]. Most of malignant insulinomas are sporadic (about 97%), even if a few cases of association with MEN1 and 1 case of association with type-1 neurofibromatosis have been described [8].

In affected patients, the control of glycemia before surgical excision, or for a prolonged time if surgery is not feasible, may be very problematic. Admittedly, the description of new cases may be of help for clinicians, who have to deal with similar situations. We report a case of malignant insulinoma associated with local infiltration, neoplastic thrombosis and lymph node metastasis, in which medical treatment with octreotide effectively counteracted hypoglycemia before surgery. One peculiarity of this case is represented by the fact that short acting octreotide was used, in agreement with the surgeon, in order to avoid any possible pharmacological interference caused by long acting formulations at the time of surgery, which was performed shortly after the diagnosis.

## Case presentation

A 68-year-old man presented a weight increase of 7 kg during the last year and symptoms compatible with hypoglycemia (objective vertigo, feeling of an empty head, sweating, palpitations). During some of these episodes low blood glucose levels (< 40 mg/dl) were documented by glucometer measurement. The symptoms regressed with food intake. These episodes initially occurred during physical activity and later also during fasting.

He had no family history of endocrine disease.

At admission, his body mass index was 28 kg/m^2^. The rest of the physical examination was unremarkable. Biochemical assessment did not show any abnormality, and glucose level was 70 mg/dl (n.v. 65–110). Plasma cortisol at 8 a.m. was in the normal range (394.8 nmol/l, n.v. 138–685 nmol/l), anti-insulin antibodies were negative, chromogranin A was 69 ng/ml (n.v. 10–185), prolactin was 247 mU/l (n.v. 53–369), gastrin was 12.7 pg/ml (n.v. < 180), PTH was 6.4 pmol/l (n.v. 1–6.8) and serum calcium level was 8.8 mg/dl (n.v. 8.6–10.4).

The fasting test was performed, which was interrupted after 12 h due to the onset of symptomatic hypoglycemia (44 mg/dl, glucometer measurement). Plasma glucose level was 41 mg/dl, insulin level 16.3 U/L and C-peptide 1.27 nmol/l (Table 1). Per protocol, 1 mg of glucagon was injected intravenously after interruption of the fasting test and plasma glucose was measured (time 0′, 41 mg/dl; after 10 min 75 mg/dl, after 20 min 94 mg/dl and after 30 min 93 mg/dl). The patient’s laboratory results showed endogenous hyperinsulinemia, according to published guidelines (fasting test: plasma glucose < 55 mg/dl, with insulin and C-peptide levels > 3 U/L and > 0.2 nmol/L, respectively; glucagone test: > 25 mg/dL increase of glucose levels after fasting) [9]. The short octreotide test (subcutaneous infusion of 100 mg of short-term octreotide at 7.00 a.m. after an overnight fast, and blood glucose, insulin and C-peptide hourly sampling for six hours) was performed to evaluate the efficacy of a possible treatment with somatostatin analogs, in order to counteract hypoglycemia [10]. No food was allowed during the test. The test showed an increase in plasma glucose above 100 mg/dl (Table 2), and the patient was considered to be responsive to somatostatin analogs [10].

**Table 1:** Fasting test results. The test has been performed according to the Endocrine Society Clinical Pratice Guidelines on adult hypoglycemic disorders (ref [9])

	12 a.m.	2 a.m.	4 a.m.	8 a.m.	10 a.m.	12 p.m.
Glucometer measurement mg/dL	111	68	67	66	62	45
Glycemia (basal 65–110 mg/dL)		70		58		41
Insulin level (basal 3–17 U/L)		8.7		13.3		16.3
C-peptide (basal 0.37–1.47 nmol/L)		1.11		1.03		1.27

**Table 2:** Short term octreotide test. The test was performed according to ref. [2]

	0’	1 h	2 h	3 h	4 h	5 h	6 h
Glycemia (basal 65–110 mg/dL)	54	61	116	134	133	130	122
Insulin level (basal 3–17 U/L)	16.8	2.5	3.1	4.3	4.5	5.2	6
C-peptide (basal 0.37–1.47 nmol/L)	1.39	0.56	0.49	0.51	0.54	0.56	0.59

**Fig. 1:**
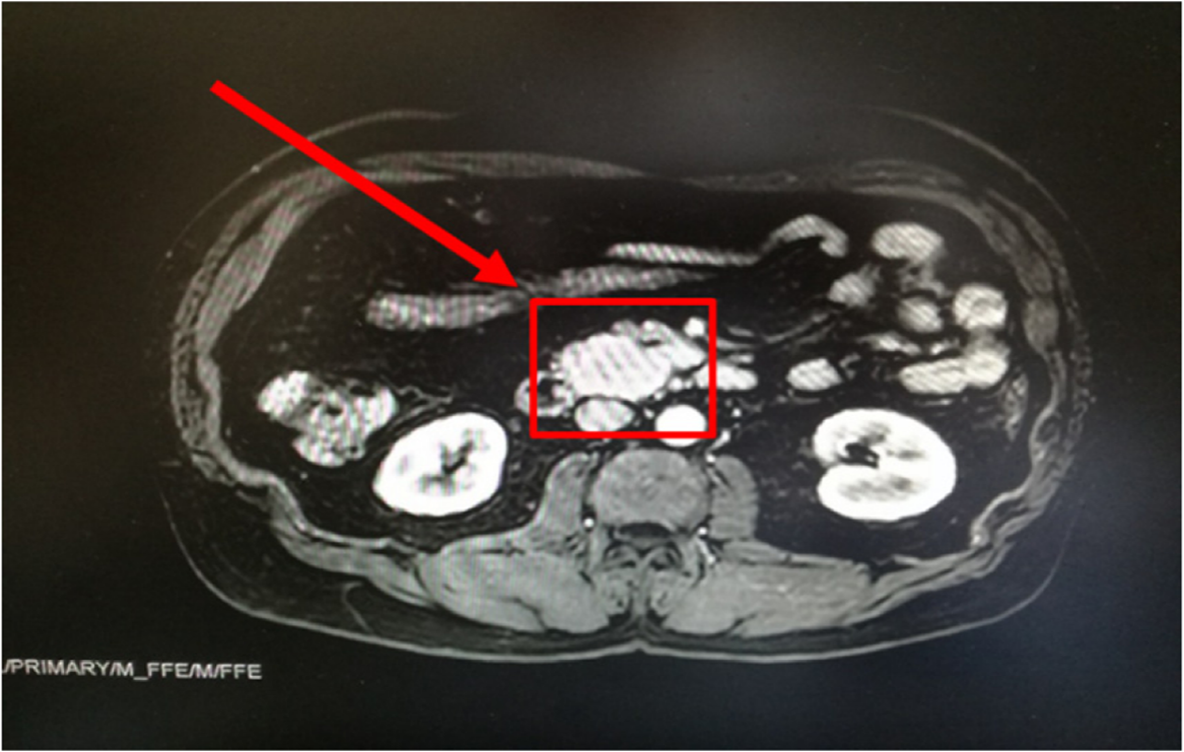
Abdominal MRI showing a solid tumor with definite margins of the uncinate pancreatic process of 4x3x3.5 cm

**Fig. 2:**
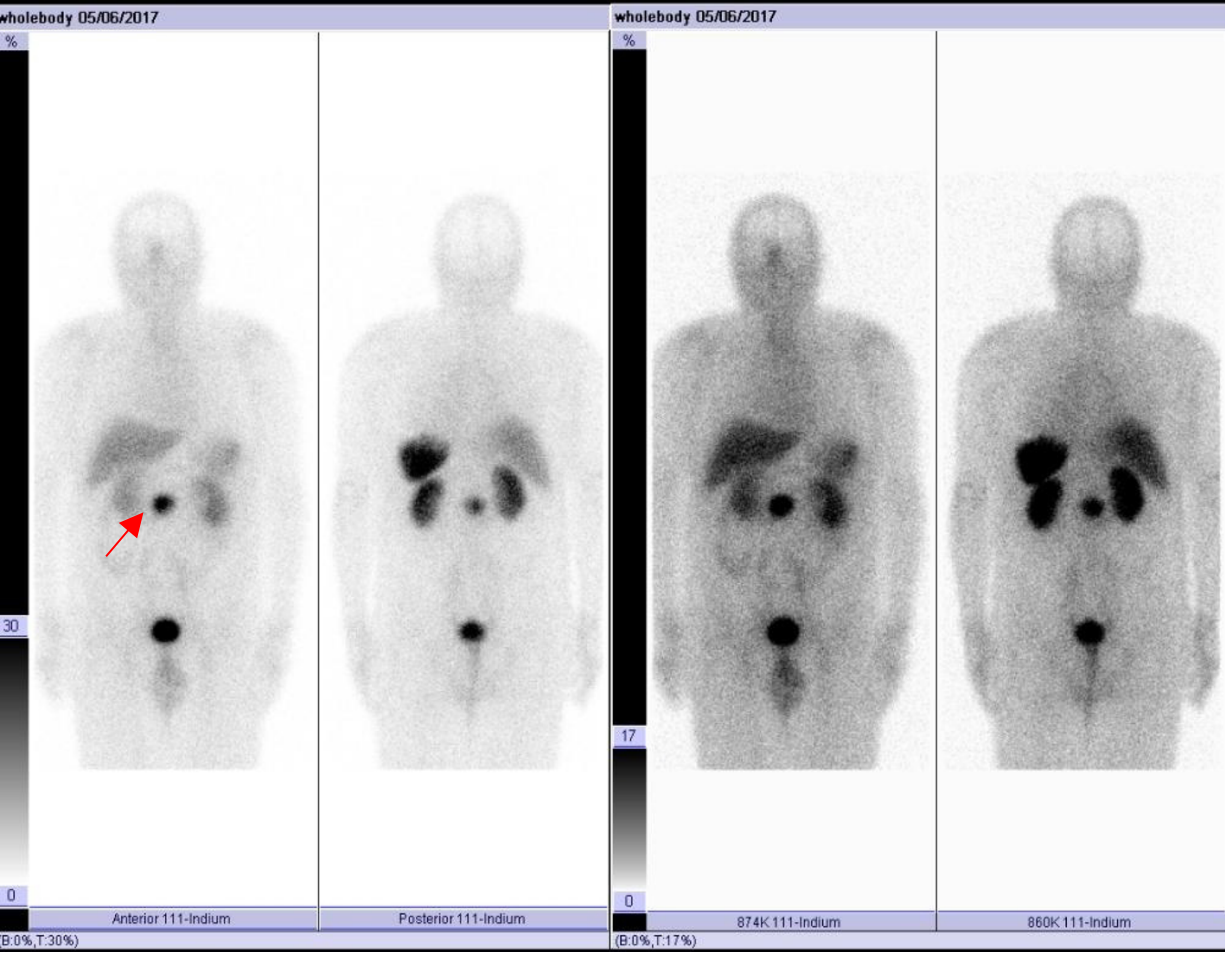
Octreoscan showing a lesion localized in the pancreatic site, with high sstR density

Additional procedures included:

- MRI, which revealed a lesion of the uncinate pancreatic process of about 4 cm, which presented a brief contact with the upper mesenteric artery and a > 180° contact with the superior mesenteric vein with suspected infiltration (Fig. 1).
- Octreoscan, which showed a lesion located in the pancreatic site, with a high somatostatin receptor (sstR) density (Fig. 2).
- Abdominal CT scan with contrast, which revealed a polylobed lesion with sharp margins at the level of the uncinate pancreas process, in contact with the superior mesenteric vein and reaching the margins of the superior mesenteric artery. No signs of vessels infiltration were observed.

All these imaging procedures were indicative of the presence of an insulinoma as the cause of endogenous hyperinsulinemia.

Surgical treatment was scheduled. Before surgery, considering the response to the short octreotide test, the patient was treated with short acting octreotide (0.1 mcg twice a day), obtaining a good control of glycemia by glucometer measurement, disappearance of hypoglycemic symptoms and no recurrence of hypoglycemic episodes.

The patient underwent exploratory laparotomy and duodenocephalopancreasectomy after one month.

The postoperative course was complicated by the appearance of a pancreatic (grade B) [11] and a biliary fistula, whereby the patient was conservatively treated with fasting and parenteral nutritional intake and subsequenty via a naso-enteral tube. The control CT scan, performed a month later, did not reveal intra-abdominal spill and drainages were removed. Oral feeding was resumed one month after the operation.

**Fig. 3:**
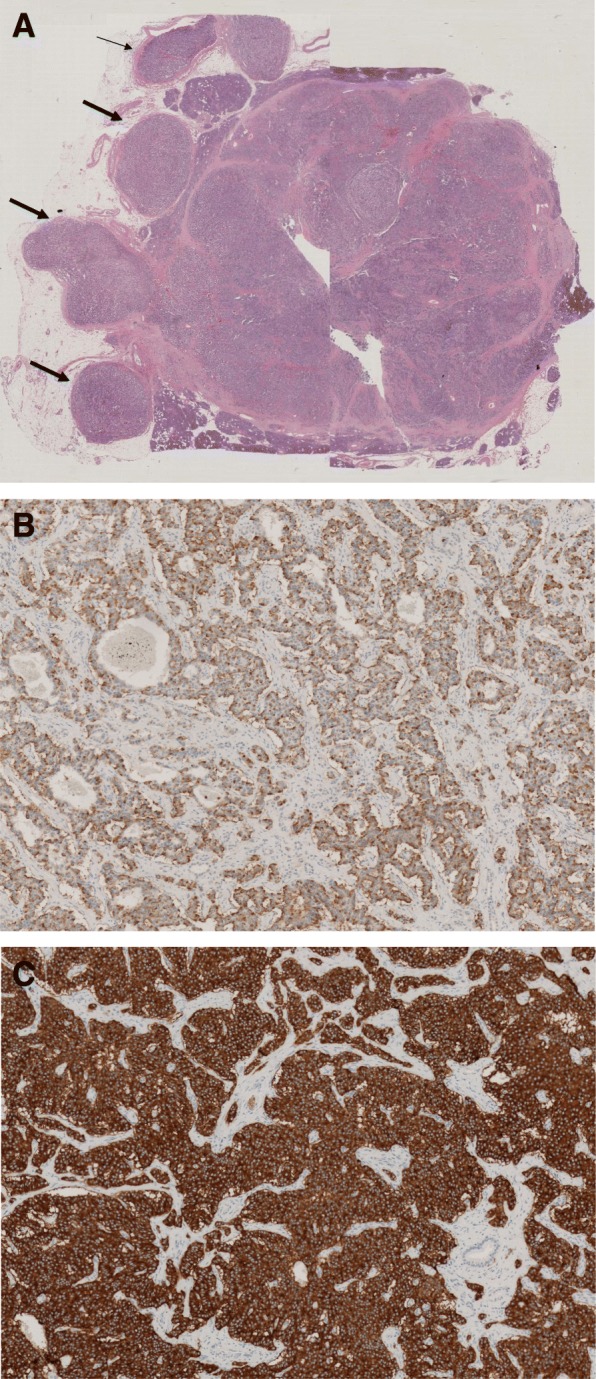
**a** Macrosection of the neoplasia showing vascular (thin arrow) and adipose tissue (thick arrows) infiltration by the tumor. **b**-**c** Immunohistochemical staining for insulin and synaptophysin, respectively

The definitive histological examination revealed a neuroendocrine tumor with insulin receptors (insulinoma) of 3.5 cm infiltrating the duodenum, retroperitoneal adipose tissue, with widespread neoplastic thrombosis, and with metastasis in 3 pancreatic-duodenal lymph nodes among the 48 that had been surgically removed (T3N1MO, AjCC VIII edition G1), with a low replicative index (Ki67: 2%); radical resection (R0). Vascular and adipose tissue infiltration by the tumor, which are indicative of malignancy, were observed. Immunohistochemical staining was positive for insulin and synaptophysin (Fig. 3).

The most recent abdominal MRI, performed 6 months after surgery, did not reveal any recurrence of disease and currently the patient is in good conditions and with normal glycemic levels.

## Discussion and conclusions

Clinical hypoglycemia occurs when plasma glucose concentration is low enough to cause symptoms and/or signs, which include neurological alterations. The clinical feautures of hypoglycemia are non specific and it is not possible to establish a single plasma glucose concentration that definitively confirms clinical hypoglycemia. Therefore, hypoglycemia is confirmed by the documentation of the Whipple’s triad [12], e.g. symptoms and/or signs consistent with hypoglycemia, a low plasma glucose concentration, and resolution of clinical alterations after the plasma glucose concentration is increased [12, 13].

The most common cause of hypoglycemia is represented by insulin, insulin secretagogues, alcohol abuse and drugs of different classes [14]. Hypoglycemia may occur during sepsis and other critical illnesses, which include renal or hepatic failure. Hypoglycemia can be also secondary to cortisol deficiency [15]. It is therefore reasonable to assess plasma cortisol in the presence of hypoglycemia, although adrenocortical failure is not a common cause of hypoglycemia in adults in the absence of other clinical evidence. A low plasma cortisol concentration found in the presence of hypoglycemia is not per se indicative of adrenocortical insufficiency. In fact, recurrent hypoglycemia shifts glycemic thresholds for cortisol secretion [16].

Hypoglycemia may be caused by hyperinsulinism in the absence of prior gastric surgery or after Roux-en-Y gastric bypass for obesity [15]. It can also be associated to the presence of anti-insulin antibodies, such as in Hirata syndrome [17]. Finally, hyperinsulinemic hypoglycemia may be due to uncontrolled insulin release either from tumoral pancreatic beta-cells or from functionally defective beta-cells, as observed in nesidioblastosis, which is usually seen in newborns [18].

Although rare, insulinoma is the most common neuroendocrine tumor of the pancreas with an annual incidence of four in every 1 million persons. Malignancy is observed in only 10% of cases [2]. The clinical manifestation of insulinoma are variable and nonspecific and are related to the presence of hypoglycemia. The symptoms, which are often precipitated during physical exercise, become typically evident after fasting. The 72-h fasting test remains the gold standard for the diagnosis of insulinoma and includes the measurement of plasma glucose, insulin, C-peptide, at the time hypoglycemic symptoms appear [19]. We used this test for the diagnosis of primary hyperinsulinism in our case and the severe hypoglycemia with measurable insulin levels occurred after only 12 h unequivocally confirmed the hypothesis.

Non-invasive imaging procedures, such as CT and MRI, are used when a the biochemical diagnosis of primary hyperinsulinism has been made. Invasive modalities, such as endoscopic ultrasonography and arterial stimulation venous sampling, have frequently been shown to be superior to non-invasive localization techniques to preoperatively localize insulinomas [19]. Numerous studies have shown that the cell surface in neuroendocrine tumors (NETs) express sstR and have led to the development of new localization techniques. 11In-[DTPA-D-Phe1] octreotide scintigraphy (Octreoscan) can be used for the localization of primary tumours and their metastases in patients presenting with the clinical and biochemical features of NETs [20]. However only 20–50% of insulinoma can be dectected by octreoscan with planar imaging [10, 21, 22], although it has been described that the use of SPECT improves the detection of insulinomas by octreoscan scintigraphy [23]. GLP-1R imaging by ^111^In-DOTA-exendin-4 administration is another non-invasive diagnostic approach that may succesfully localize small insulinomas pre- and intraoperatively [24]. Very recently, the ENETS guidelines recommend ^68^Ga-DOTA-somatostatin analog PET/CT, because it is largely superior to somatostatin receptor scintigraphy, and facilitates the diagnosis of most types of NET lesions [25]. In our patient MRI showed the presence of a lesion of the uncinate pancreatic process of about 4 cm. Subsequent octreoscan revealed that the lesion expressed high levels of sstR.

Surgery is the first choice therapy for resectable insulinomas. A pharmacological approach can be useful both during the preoperative period, and for preventing hypoglycaemia in insulinomas with unknown localization. Diazoxide can prevent hypoglycemia by suppressing the release of insulin from insulinoma cells via opening ATP-sensitive potassium channels [26]. The use of this drug may be limited by side effects, such as hypotension, water retention with declining edema, hyperuricaemia, hypertriglyceridaemia, thrombocytopenia and neutropenia. Somatostatin analogs represent another possible medical strategy to suppress uncontrolled insulin secretion and control the symptoms of hypoglycemia in patients with insulinoma [27]. They can be used for instance in patients who are not eligible for surgery and when diazoxide is not applicable due to its inefficiency or adverse effects. In malignant insulinomas the use of somatostatin analogs may have an additional indication, due to the antiproliferative and moderate antineoplastic activity of these molecules [28–31].

It has to be said that the response to somatostatin analogs may differ according to the presence of various subtypes of sstR on insulinoma cells. Octreotide binds predominantly to sstR subtype 2. The absence of these receptors on insulinoma cells may aggravate hypoglycemia when a patient is treated with octreotide. This effect may be due to the inhibition of contra-insular hormones such as growth hormone and glucagon by somatostatin [32, 33]. The glycemic response to somatostatin analogs in any single patient is unpredictable and for this reason testing the drug while the patient is hospitalized is mandatory. In the case we described, a very good response was obtained with octreotide administration and the patient did not experience any new hypoglycemic episode before surgery.

In summary, we have described a rare case of malignant insulinoma in a patient with recurrent hypoglycemic episodes. The patient was successfully treated with short acting octreotide analogs before surgery, after testing its efficacy on glycemic control. No disease recurrence was observed at 6 month after surgery and the condition of the patient is currently very satisfactorily.

## Abbreviations

CT scan: Computed Tomography scan
MEN-1: Multiple Endocrine neoplasia-1
MRI: Magnetic Resonance Imaging
PTH: Parathyroid hormone
